# Tribocorrosion Behavior of NiTi Biomedical Alloy Processed by an Additive Manufacturing Laser Beam Directed Energy Deposition Technique

**DOI:** 10.3390/ma15020691

**Published:** 2022-01-17

**Authors:** Mihaela Buciumeanu, Allen Bagheri, Filipe Samuel Silva, Bruno Henriques, Andrés F. Lasagni, Nima Shamsaei

**Affiliations:** 1 Department of Mechanical Engineering, Faculty of Engineering, “Dunărea de Jos” University of Galaţi, Domnească 47, 800008 Galati, Romania; 2 Center for Advanced Vehicular Systems (CAVS), Mississippi State University, Starkville, MS 39762, USA; bagheri.274@gmail.com; 3 Center for Micro-Electro Mechanical Systems (CMEMS-UMinho), Campus de Azurém, University of Minho, 4800-058 Guimarães, Portugal; brunohenriques@dem.uminho.pt; 4 Laboratory of Ceramic and Composite Materials (CERMAT), Campus Trindade, Federal University of Santa Catarina (UFSC), Florianópolis 88040-900, SC, Brazil; 5 Institute for Manufacturing Technology, Technische Universität Dresden, 01062 Dresden, Germany; andres_fabian.lasagni@tu-dresden.de; 6 Fraunhofer-Institut für Werkstoff- und Strahltechnik IWS, Winterbergstr. 28, 01277 Dresden, Germany; 7 Department of Mechanical Engineering, Auburn University, Auburn, AL 36849, USA; shamsaei@auburn.edu; 8 National Center for Additive Manufacturing Excellence (NCAME), Auburn University, Auburn, AL 36849, USA

**Keywords:** NiTi, Ti-6Al-4V, laser engineered net shaping (LENS), tribocorrosion

## Abstract

The purpose of the present study was to experimentally assess the synergistic effects of wear and corrosion on NiTi alloy in comparison with Ti-6Al-4V alloy, the most extensively used titanium alloy in biomedical applications. Both alloys were processed by an additive manufacturing laser beam directed energy deposition (LB-DED) technique, namely laser engineered net shaping (LENS), and analyzed via tribocorrosion tests by using the ball-on-plate configuration. The tests were carried out in phosphate buffered saline solution at 37 °C under open circuit potential (OCP) to simulate the body environment and temperature. The synergistic effect of wear and corrosion was found to result in an improved wear resistance in both materials. It was also observed that, for the process parameters used, the LB-DED NiTi alloy exhibits a lower tendency to corrosion as compared to the LB-DED Ti-6Al-4V alloy. It is expected that, during the service life as an implant, the NiTi alloy is less susceptible to the metallic ions release when compared with the Ti-6Al-4V alloy.

## 1. Introduction

Titanium alloys are one of the most commonly used metallic materials in biomedical applications [1,2,3]. As materials for implants, they are subject to synergistic interactions between mechanical and corrosive actions [3]. It is well known that all titanium alloys have the ability to form a protective layer in the presence of a bodily fluid (saliva, phosphate buffered saline, etc.), which may somewhat protect them from corrosion [2,4]. While the protective layer can delay/avoid the dissolution of the metallic ions in these conditions (without any mechanical action), this layer may be removed under mechanical loading, thus leading to the release of the metallic ions. The degradation caused by the combined effects of mechanical and electrochemical actions, the so-called tribocorrosion, is generally completely different from these failure mechanisms acting separately.

In spite of good properties of titanium alloys, such as lower Young’s modulus, high strength, good workability, biocompatibility, corrosion resistance, and more, the major concerns still remain regarding wear resistance and also the release of the metallic ions during the service life of a titanium implant in the human body [5,6]. There are many studies published in the technical literature concerning the wear and corrosion, as well as the tribocorrosion behavior, of Ti-6Al-4V alloy [4,7,8]. As reported in the clinical studies, a higher metallic content released into the human body leads not only to the failure of the implant (i.e., loosening) but also to opposing biological reactions, such as toxicity and allergies [5,6,9,10].

Nowadays, new smart materials are being considered to produce smart structures in biomedical engineering. However, their performance first needs to be thoroughly characterized [11,12]. NiTi alloy is used as a biomedical material (e.g., archwire for tooth positioning in dental applications, surgical tools, and stents) [1,13,14,15] due to its unique shape memory, pseudoelasticity, or superelasticity properties. In comparison with Ti-6Al-4V alloy, NiTi alloy has a better biocompatibility, as well as lower stiffness and damping capacity [16].

Thus, both the Ti6Al4V and NiTi alloys considered in this study are used in medicine, but for different applications. Therefore, it is interesting to see whether Ti6Al4V alloy can be substituted by NiTi alloy in some medical uses, such as implants. It is well known that the materials used for implants should have some fundamental technical specifications. There should be a compromise between the bulk and surface properties (lower Young’s modulus that should be comparable to the bone, high strength, adequate ductility, high hardness, etc.), and, of course, biocompatibility (corrosion resistance, etc.) [17]. The major concern in the failure of implants is related to the bone in-growth process after the implantation [18]. Therefore, NiTi alloy, due to its good wear resistance and shape memory effect, may act as a key factor in the healing process by stimulating the bone in-growth properties [19,20] and thus reducing the healing time and, in some cases, avoiding a new surgery.

Considering the fact that NiTi alloy is very challenging to be machined, various processing methods, such as casting and powder metallurgy processes, as well as more advanced techniques, such as additive manufacturing, are often employed to produce NiTi parts, specifically when it is necessary to have complex geometries such as the ones of implants [21]. By traditional processing technologies, such as casting, it was only possible to obtain NiTi components with very simple forms, such as wires, rods, and sheets [20]. The difficulty to produce complex NiTi parts by casting technology was mainly attributed to the formation of impurities (carbon and oxygen contamination) and the Ti-rich phases due to the high melting temperature required during the process. As mentioned by Mehrpouya and co-authors, the Ni content is the chemical element that affects the phase transition temperatures [22].

Another method used to produce complex components is powder metallurgy (e.g., hot pressing). By this method, it is possible to produce components with an almost finished shape, thus reducing the necessity of extensively machining, as in the case of casting technology. Despite some advantages of hot pressing technology, such as less material discarded, less energy used, less sensitivity to the chemical composition change, etc., there are still problems to obtain such complex structures used in biomedical applications [4,23,24].

The additive manufacturing techniques (e.g., selective laser sintering—SLS, selective laser melting—SLM, laser engineered net shaping—LENS, electron beam melting—EBM, etc.) have been considered for the manufacturing of bio-implants due to their unique benefits, including the capability to produce tailored parts with complex geometries [4,24,25,26,27,28,29].

It has already been shown that it is possible to obtain an improved wear and corrosion resistance for Ti-6Al-4V parts produced by laser engineered net shaping (LENS) relative to the ones produced by casting or hot pressing (HP) [4]. The enhancement was explained based on a higher hardness and slight chemistry variation due to the high cooling rates experienced during LENS processing (10^3^–10^5^ K/s). Despite the increase in the price of the components processed by the additive manufacturing technologies (3D or 4D printing), it seems that, in this way, it is possible to use most of the potential of the shape memory alloys.

There are several corrosion and/or tribological studies on NiTi alloy, and NiTi properties have been extensively compared to other materials used in medical applications, such as Ti and its alloys, 316L stainless steel, etc. [21,29,30,31,32,33,34,35,36,37]. While there are several studies dealing with the tribocorrosion behavior of NiTi alloy fabricated by traditional manufacturing methods (casting, wrought, etc.), the literature is very scarce when it comes to the tribocorrosion characterization of additively manufactured NiTi [1,15,38,39]. It has been reported by Kosec et al. [38] that the wear of NiTi increased in the presence of artificial saliva and the corrosion was accelerated due to the action of mechanical loading.

In the present research, the synergistic effects of wear and corrosion in phosphate buffered saline on as-printed NiTi shape memory alloy processed by LB-DED LENS have been investigated. This paper reports our research on the tribocorrosion behavior of NiTi alloy in comparison to the most extensively used titanium alloy (i.e., Ti-6Al-4V) in biomedical applications manufactured by the same processing method (i.e., LB-DED LENS).

## 2. Materials and Methods

### 2.1. Materials and Sample Preparation

NiTi alloy spherical powder particles, (Carpenter Technology Corporation, USA) with the particle size distribution from 30 μm to 150 μm (D50 = 66.3 μm and D90 = 110 μm) were employed as the raw material for manufacturing the NiTi samples [26]. As can be seen from Table 1, the chemical composition of the powder included 55% nickel and 43% titanium in weight percent (50.7% Ni–48.6%Ti in atomic percent).

Ti-6Al-4V alloy spherical powder particles with the mean particle size of 32.53 μm with the following chemical composition were used (as provided by TLS Technik, Germany, in weight %): Al 6.4, V 3.8, C 0.01, Fe 0.23, O 0.12, N 0.02, H 0.0074, and balance Ti was employed to fabricate the LB-DED Ti-6Al-4V samples.

NiTi and Ti-6Al-4V samples were produced by using a 750 LENS^®^ machine (Optomec, Inc., St Paul, MN USA). The LENS process parameters utilized in manufacturing the samples, such as powder feed rate, traverse/scan speed, laser output power, layer thickness, and hatch spacing, are listed in Table 2. These parameters used to produce Ti6Al4V and NiTi samples were selected based on previously carried out process optimization studies [26,40,41,42], which have shown that the process parameters presented in Table 2 led to the best mechanical properties. NiTi and Ti-6Al-4V samples were deposited according to the following procedure: the first layer was deposited at 0° laser scanning direction, then the next layer at 90°, and so on till a rod of 7 mm diameter and 77 mm height had been obtained.

For tribocorrosion tests, samples of 3 mm height from the middle section of the produced rods were cut [26,40,43].

Each sample was polished by SiC papers down to 4000 mesh. Then, they were polished by diamond paste (1 µm) and cleaned ultrasonically by alcohol, followed by distilled water.

### 2.2. Tribocorrosion Measurements

**Figure 1:**
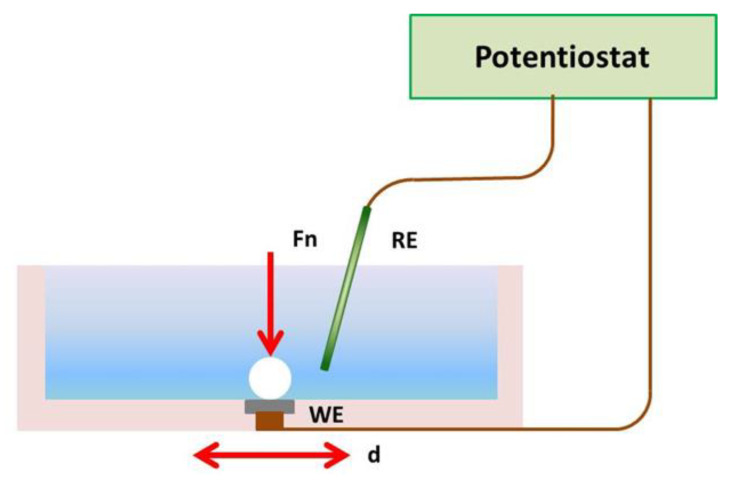
Schematic representation of reciprocating ball-on-plate tribocorrosion experimental setup: Fn—normal load, d—alternative displacement; WE—working electrode (tested sample); RE—reference electrode (saturated calomel electrode).

Figure 1 shows a schematic representation of the reciprocating ball-on-plate tribocorrosion test set-up used in this study. The reciprocating ball-on-plate configuration was selected based on the fact that one of the demands for implant materials is the necessity to endure the relative sliding motion [44] and also because it is one of the most used configurations in tribocorrosion set-ups [4,37,45].

It employed a Bruker tribometer (UMT-2–Bruker, MA, USA), a Gamry Reference 600 potentiostat/galvanostat (Warminster, PA, USA), and an acrylic electrochemical cell (attached to the tribometer, serving as holder for the tested samples and also as bath for the electrolyte).

The produced LB-DED NiTi and Ti-6Al-4V samples were used as plates, while an alumina 10 mm ball (Goodfellow) was used as counterface as this is a non-conductive material. During the test, the alumina and LB-DED metallic sample tribocontact is continuously immersed into a phosphate buffered saline (PBS) solution. The composition of the PBS solution is given in a previous work [4].

For the electrochemical measurements, a two-electrode cell with a saturated calomel electrode (SCE) as the reference electrode (RE) and the metallic LB-DED samples as working electrode (WE) was used. As can be seen from Figure 1, the RE is immersed in PBS and placed very close to the WE. All tribocorrosion tests were carried out under open circuit potential (OCP), which means that, during the tests, no voltage was applied. OCP is the potential that takes place between the tested sample and PBS solution. The OCP is measured with respect to the SCE.

The tribocorrosion test consisted of three steps:

(1) OCP measurement before sliding (passivation step). Before each reciprocating sliding wear test, the OCP was measured in situ for a minimum of 60 min.
(2) OCP measurement during the reciprocating sliding wear test (removal of the passive layer step). The reciprocating wear sliding tests were performed at 1 N normal load, 1 Hz, and at the 3 mm total stroke length for 30 min. Both the OCP and coefficient of friction (COF) were measured during the sliding.
(3) OCP measurement after sliding (repassivation step). At finishing the sliding tests, the OCP was measured for another 60 min.

All tests were carried out at normal human body temperature of 37 ± 2 °C. One set of each test condition was repeated five times. More details regarding the tribocorrosion tests are given in a previous work of the authors [46].

### 2.3. Surface Characteristics

For microstructural characterizations and wear tracks analyses, the LB-DED samples were examined using SEM/EDS (scanning electron microscopy/energy dispersive spectrometer—JSM-6010Lv, Jeol USA Inc., Peabody, MA, USA).

X-ray diffractometer (XRD) using Bruker AXS D8 Discover (Bruker, Karlsruhe, Germany) was used to identify the phase of the LB-DED NiTi and Ti-6Al-4V samples. XRD was performed in the 2-theta (2θ) range between 30–90° with a Cu-Kα radiation source, 0.05° scan step, and a 2 s counting time.

For analyzing the surface topography of the wear tracks, a confocal microscope (Sensofar S Neox, Barcelona, Spain) with a 150× objective was used, resulting in vertical and lateral resolution of 2 nm and 140 nm, respectively.

## 3. Results and Discussion

### 3.1. Microstructural Characterization and Phase Analysis

**Figure 2:**
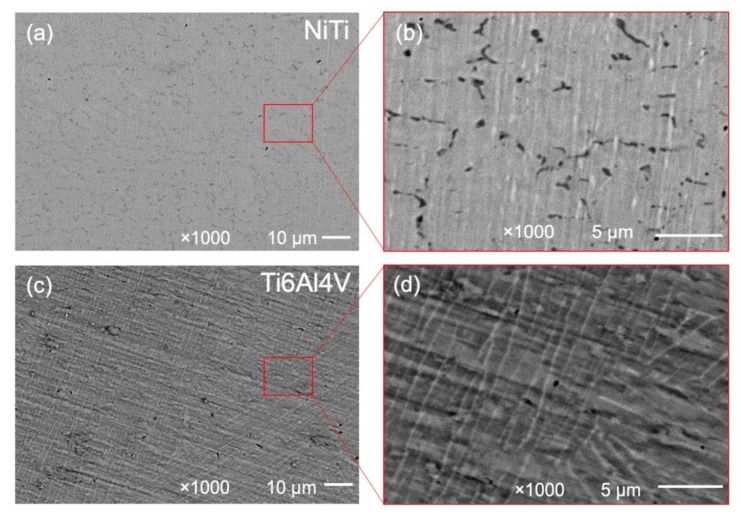
Microstructure of LB-DED NiTi (**a**,**b**) and Ti-6Al-4V (**c**,**d**) alloys with two different magnifications (1000× and 5000×).

**Figure 3:**
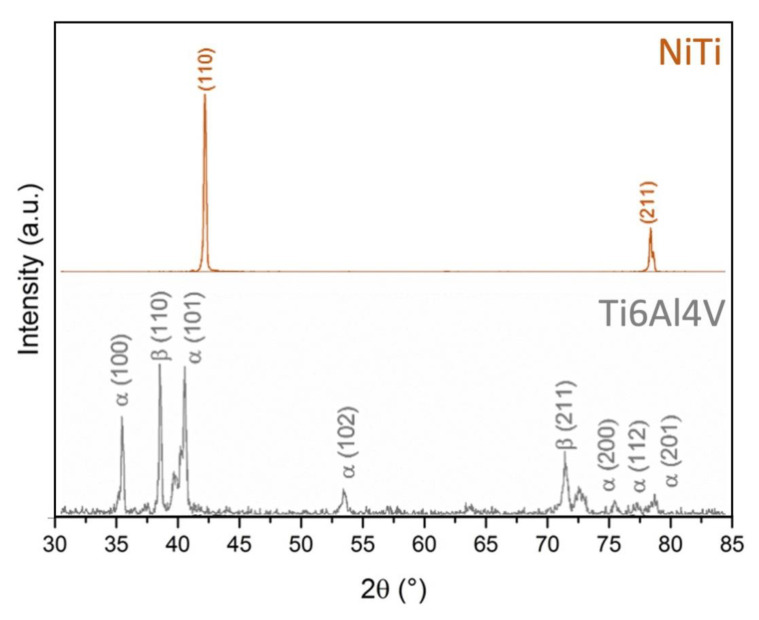
XRD spectrum results of LB-DED NiTi alloy (**top** row) and Ti-6Al-4V alloy (**bottom** row) [4].

The SEM micrographs showing the microstructures of the LB-DED NiTi and Ti-6Al-4V alloys at two different magnifications (1000× and 5000×) are shown in Figure 2, while the X-ray diffraction spectra of the LB-DED NiTi and Ti-6Al-4V alloys are shown in Figure 3.

Typically, in the case of materials manufactured by additive technique (by selecting the appropriate parameters), finer microstructures are formed due to the higher cooling rates [47]. As a result, increased mechanical strength and enhanced wear resistance are typically obtained [47]. Furthermore, the cooling rate has a major influence on the microstructure evolution, and the LENS process is well known for the high cooling rates (10^3^ to 10^5^ K/s) [40,48].

The microstructure of the LB-DED NiTi alloy is revealed in Figure 2a. The average grain size of this alloy, as achieved through the LENS process, is about 15 μm [26]. Our data are in accordance with the findings of Marattukalam et al. [28], which report comparable values for the grain size (8–16 μm) using the same processing technology. Among all the parameters used to produce LENS samples, as reported by Marattukalam, the laser power seems to be the major factor that influences the grain size of the NiTi alloy. The grain growth with increasing laser power was explained based on the rise in laser energy input, and, at the same time, it was linked with the reduced cooling rate.

The microstructure of the LB-DED Ti-6-Al-4V alloy is given in Figure 2b and reveals large columnar prior β grains [40].

The XRD spectrum of the LB-DED NiTi alloy (Figure 3—top row) indicates only two visible phases: a strong peak of austenite phase-B2 110) and a much weaker peak of martensitic phase-B19 (211). The laser energy density is the parameter that has a great influence on the variation of the ratio of the austenite and martensitic phases [49]. Thus, the higher amount of B2 phase observed in this work may be explained based on the rapid solidification rates due to the laser energy density used (66.1 J/mm^2^) to produce the TiNi samples.

The XRD spectrum of LB-DED Ti-6Al-4V (Figure 3—bottom row) shows the presence of α phase with a higher concentration and a lesser concentration in β phase. Details regarding the LB-DED Ti-6-Al-4V microstructure and also about the XRD spectrum are given in [4].

### 3.2. Wear Volume Loss and Coefficient of Friction (COF) Measurements

**Figure 4:**
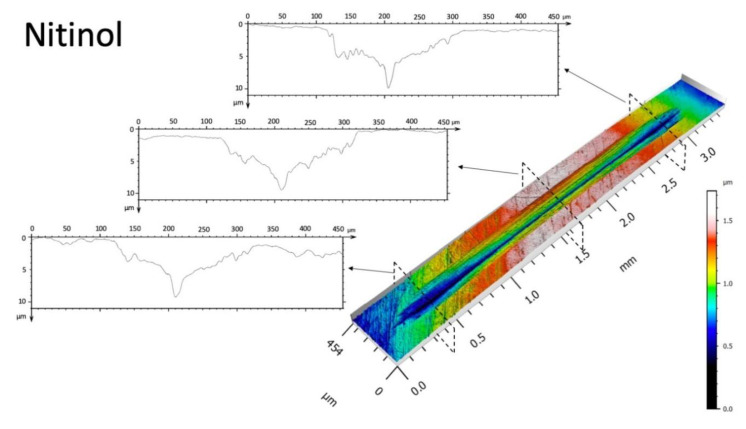

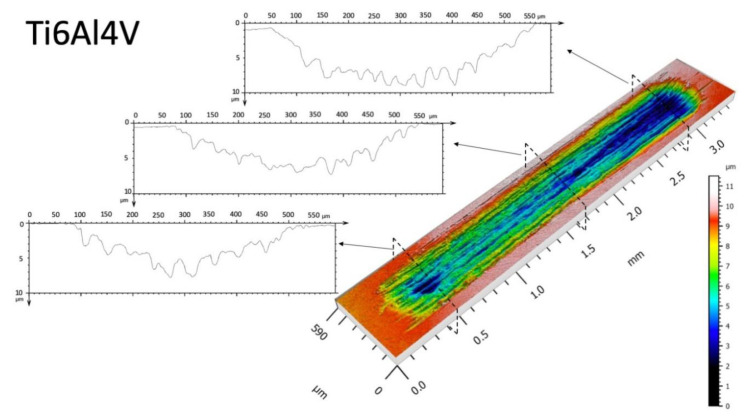
Surface topography images of the wear tracks and also the worn surface profiles (three different zones) for LB-DED NiTi and Ti-6Al-4V alloys.

**Figure 5:**
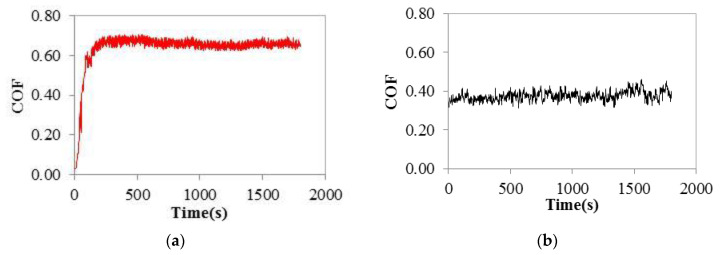
Evolution of COF with sliding time for LB-DED (**a**) NiTi and (**b**) Ti-6Al-4V alloys.

Figure 4 shows the surface topography images of the wear track for the LB-DED NiTi and Ti-6Al-4V alloys and also three 2D profiles in different zones of the wear tracks, while the evolution of the COF with time is given in Figure 5.

It can be clearly seen that the wear track profile in the case of the LB-DED NiTi alloy has a completely different shape compared to the one of the LB-DED Ti-6Al-4V alloy. The wear volume losses of the LB-DED NiTi and Ti-6Al-4V alloys were calculated from the wear track profiles obtained by confocal microscopy.

The LB-DED NiTi alloy exhibits a wear volume loss with an average value of 2.03 × 10^−4^ mm^3^ (Figure 4). This value is much lower than the wear volume loss value obtained for the LB-DED Ti-6Al-4V alloy fabricated by the same processing technology, which was measured to be 5.02 × 10^−3^ mm^3^. In the present study, the hardness is not in perfect correlation with the wear results. In the case of the LB-DED NiTi alloy, the hardness value is lower (338 HV ± 9) than the value obtained for the LB-DED Ti-6Al-4V alloy, namely 415 HV ± 2. In the technical literature, the wear resistance of NiTi alloy when the material is in contact with a sliding ball was mainly correlated to the pseudoelasticity property that is influenced by the reversible martensitic transformation [30,31,32,35]. Regardless of the processing technology, the low wear volume loss exhibited by the additive manufactured NiTi alloy in this study is in good agreement with the high wear resistance results reported in the literature [30,31,35,37,50]. For example, Zhang et al. [35] reported a wear resistance of cast NiTi alloy (using ball-on-disc configuration) that was 30 times higher than of pure Ti under dry sliding conditions.

Abedini et al. [31] studied the tribological behavior of a wrought NiTi alloy under a wet environment in two different states: martensitic and austenitic. In the austenitic state, the NiTi alloy exhibited better tribological behavior (high wear resistance and low COF value) as compared to the NiTi alloy in the martensitic state. The improvement obtained in the case of the austenitic state was mainly related to the pseudoelasticity effect and higher strength of the NiTi alloy.

Regarding the COF values obtained in this study, it can be seen from Figure 5 that, in the case of the LB-DED NiTi alloy, a higher value of around 0.62 ± 0.03 was registered as compared to the LB-DED Ti-6Al-4V alloy with a value of around 0.37 ± 0.01. Similar COF values recorded during the steady state stage are reported in the literature for NiTi alloy, ranging from 0.5 to 0.7 (under wet conditions) [1,38]. Concerning the evolution of COF with time, the typical two friction stages were observed: running-in and steady state stages. The running-in stage can be better observed in the case of the LB-DED NiTi alloy (Figure 5a) since it is a bit longer than in the case of the LB-DED Ti-6Al-4V alloy (Figure 5b). Thus, in both cases, the COF values increased until the steady state value was reached. Some oscillations of the COF values with sliding time could be observed in the case of the LB-DED Ti-6Al-4V alloy (Figure 5b), while a more stable evolution of the COF values is obtained in the case of the LB-DED NiTi alloy (Figure 5a). The oscillations observed in the COF evolution of the LB-DED Ti-6A-l4V alloy are associated to the release of metallic particles during sliding movement, which means that the third body effect was active (three-body abrasive wear).

### 3.3. Open Circuit Potential (OCP) Measurements

**Figure 6:**
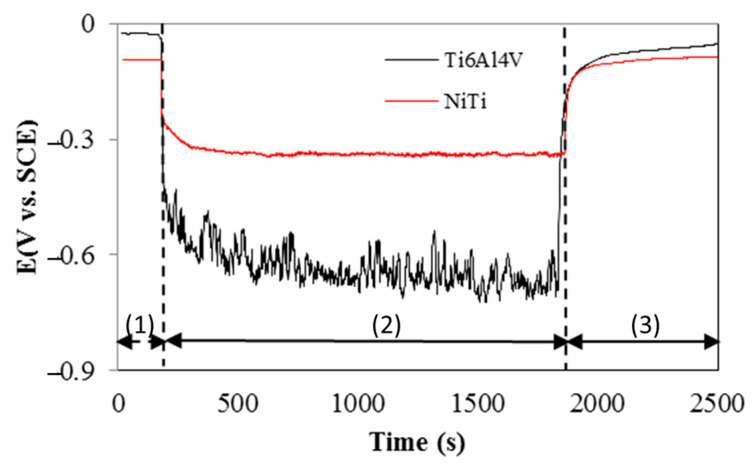
Evolution of OCP recorded (1) before sliding, (2) during the reciprocating sliding wear test, and (3) after sliding.

The evolution of the OCP recorder for all three steps of the tribocorrosion test is presented in Figure 6. The OCP was measured before sliding, when (1) no load is applied (only under the influence of corrosive environment), (2) during the reciprocating sliding wear test and, therefore, under loading (under the synergetic action between the mechanical loading and the influence of corrosive environment), and (3) after sliding, when the load is removed (only under the influence of corrosive environment).

The modifications of the OCP values before sliding (passivation step) offer important information regarding the electrochemical reactivity of the tested samples. The reciprocating sliding wear test can be started only if it has attained a steady state, which means the development of the passive oxide film on the surface of the metallic material. This state is achieved when the oscillations of the OCP values are below 1 mV/min [51]. As the necessary time to achieve a steady state varies based on the material tested and electrolyte used, the 60 min time point was selected considering the system used in this study (titanium-based alloys and PBS solution). Correspondingly, Figure 6 shows that, before the mechanical action (till time 180 s), the OCP value registered for the LB-DED Ti-6Al-4V samples was approximately −0.03 V, while, in the case of the LB-DED NiTi samples, it was approximately −0.09 V. Thus, it is possible to state that, under static corrosive conditions (no mechanical action), the LB-DED Ti-6Al-4V samples showed a slightly lower tendency to corrosion than the LB-DED NiTi samples.

However, at the moment when the normal load was applied on the alumina ball, a cathodic shift of the OCP (see the drop at time 180 s) towards more negative values was observed for both of the alloys under study. Due to the high contact pressure (Hertzian contact ball-on-plate), this drop to more active potentials indicates that the passive layer was removed at the tribological contact alumina ball/metallic sample, which means that the wear track started to take shape. As mentioned by Dalmau and co-authors [52], during the early stage of a wear test, there is a material flow causing a fast increase in the worn surface.

It can be seen from Figure 6 that the most sudden drop in the OCP value was obtained for the LB-DED Ti-6Al-4V sample (during sliding, the potential value was around −0.60 V). For the LB-DED NiTi alloy, the potential value during the sliding test was around −0.30 V. Thus, during sliding (Step 2—30 min—synergetic action between the mechanical loading and the influence of corrosive environment), the potential remains around these values. It can be observed that the OCP curve during the sliding regarding the LB-DED Ti-6Al-4V samples presented large oscillations, while, in the case of the LB-DED NiTi samples, the OCP curve presented a more stable and smoother evolution. This behavior may be related to the presence of the third-body effect (accumulation of the wear debris on the surface) in the case of the LB-DED Ti-6Al-4V alloy, and it is in perfect association with the COF evolution (see Figure 5b). On the other hand, these OCP oscillations are due to the tendency to local depassivation-passivation processes taking place in the contact [46]. In contrast, during the reciprocating movement of the alumina ball on the metallic samples, the surface does not have enough time to recover the protective layer and a fresh titanium surface is always exposed to corrosive medium. It seems that, in the case of the LB-DED NiTi samples, the absence of oscillations is probably due to the formation of a more stable protective layer in the used electrolyte, and, as a consequence, a lower removal of the top layer [8]. As stated by Zheng et al. [53], the stable protective layer on the NiTi alloy may be due to the grain refinement.

After the wear track formation, two zones with distinct characteristics can be seen on the surface exposed to the PBS solution: Zone 1—the wear track can be considered an active zone, from where the passive layer was removed due to mechanical action, and Zone 2—the one outside the wear track that is still covered with the oxide layer. As reported in previous studies [54,55], galvanic coupling may occur between these two different zones: the wear track acts as the cathode and the one covered by the passive layer acts as an anode. Thus, this galvanic coupling may lead to an intensification of the corrosion degradation in the wear track. In fact, the OCP recorded in the case of the LB-DED NiTi samples may be associated with a less intense galvanic coupling as compared to the OCP measured in the LB-DED Ti-6Al-4V samples.

At the moment when the normal load was withdrawn (see change on OCP evolution at time point 1980 s), an anodic shift of the OCP towards less negative values can be observed. It can be clearly seen that the behavior during the anodic shift of the OCP is slightly different from the cathodic shift. The increase in the OCP values is not as abrupt as when the normal load was applied. This may be associated to the repassivation process of the worn surface [55]. Moreover, it should be highlighted that, for both investigated materials, the OCP values came closer to the recorded values before the reciprocating sliding test, which means that the surfaces were able to repassivate. A similar behavior of OCP was reported by Mocnik et al. [1]. The repassivation ability of NiTi alloy was also confirmed by other researchers [1,14,29,33].

It has already been reported in the technical literature that the used electrolyte has a great influence on the passivation/repassivation process of NiTi alloy [14,33]. The corrosive behavior of NiTi was studied in different solutions, such as Hanks’ solution, Eagle’s minimum essential medium (MEM), Ringer’s solution, fetal bovine serum (FBS), phosphate buffer saline (PBS), among others [14,29,33]. Moreover, Hang et al. [14] observed a difference in the corrosion behavior of cast NiTi alloy when the tests were carried out in FBS and PBS. A more electronegative value of the OCP was reported when cast NiTi alloy was in contact with FBS as compared to PBS, and the oxide layer formed on the surface of NiTi alloy was more porous and thinner in the case of FBS in contrast with PBS. This was explained based on the fact that FBS accelerates leaching of Ni ions. Marattukalam et al. [29] tested the corrosion resistance of NiTi manufactured by LENS in Ringer’s solution and reported a strong positive correlation between the improvement in corrosion resistance and the increase in the laser power.

The low amount of the martensitic phase obtained in the present study may be responsible for the improvement in the tribocorrosion behavior of the LB-DED NiTi alloy. It has already been shown by Marattukalam et. al. that increasing it by heat treating the NiTi alloy (processed by LENS) makes it more susceptible to corrosion [49]. There are also studies that did not observe any modification on the localized corrosion resistance of cast or wrought NiTi with the martensitic phase [56,57]. These differences may come from the various cooling rates applied, which may influence the final hardness, and from the powder consolidation method employed, which can impact corrosion resistance.

### 3.4. Surface Morphologies

**Figure 7:**
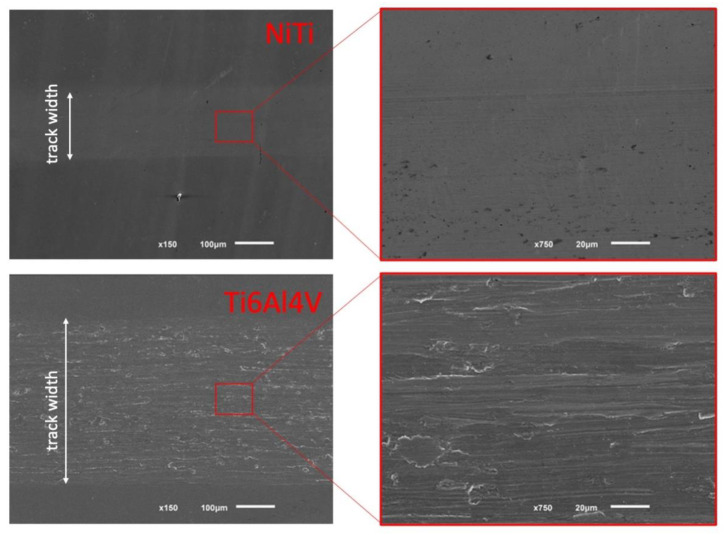
SEM images of the wear track of LB-DED NiTi alloy (**top** row) and LB-DED Ti-6Al-4V alloy (**bottom** row) [4].

In order to further understand the tribocorrosion behavior of both the LB-DED NiTi and Ti-6Al-4V alloys processed by LENS technology, the wear tracks were examined (Figure 7).

The width of the wear track in the case of the LB-DED NiTi alloy (top row) is narrower (around 210 μm), with a smoother appearance in comparison with the wear track surface in the LB-DED Ti-6Al-4V alloy (around 420 μm), indicating a lower level of surface degradation in the LB-DED NiTi samples. This observation is in accordance with the results of wear volume loss values (Figure 4) and also with the corrosive behavior (Figure 6).

Regarding the morphology of the NiTi samples, some very light grooves can be observed in the SEM image with higher magnification, shown in Figure 7-top row, which means that the predominating wear mechanism is the abrasive wear. The surface of the wear track of the LB-DED Ti-6Al-4V alloy is characterized by different wear mechanisms: abrasive wear, proved by the grooves aligned with the sliding direction due to the micro-plowing caused by debris, and slight adhesive wear, proved by some adhesive blocks and localized plastic deformation along the edges of the grooves, as seen in Figure 7-bottom row.

**Figure 8:**
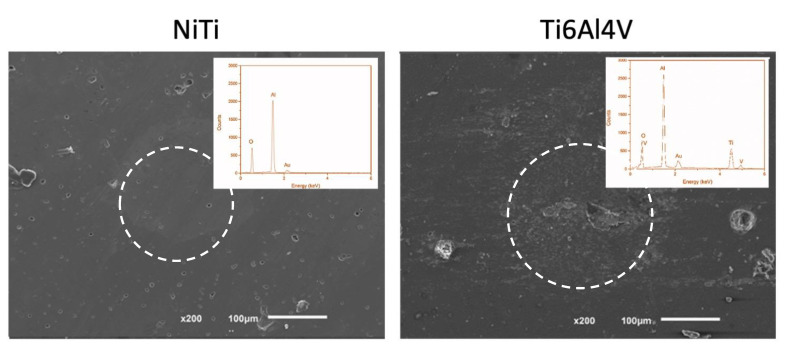
SEM images of the mating counter material surface (alumina ball) and EDS spectrum taken on the marked zones: LB-PBF NiTi alloy on top and LB-PBF Ti-6Al-4V alloy on bottom [4].

Figure 8 shows the representative morphology of the wear tracks of the mating counter material surface (alumina ball).

To identify whether any material transfer from the metallic alloys to the alumina counterface took place, an EDS analysis was performed on the marked zones. The EDS analysis confirmed the transfer of the LB-DED Ti-6Al-4V alloy to the alumina ball (presented oxygen, aluminum, titanium, and vanadium peaks), while, in the case of the alumina ball sliding against the NiTi samples, there was no evidence of it (presented only oxygen and aluminum peaks).

In summary, the tribocorrosion behavior of LB-DED NiTi alloy in the presence of PBS solution is mainly determined by abrasion of the metallic surface (of course, interacting with corrosion). This results in a low OCP value combined with a stable evolution during the test (although a higher COF value). In the case of LB-DED Ti-6Al-4V alloy, a combination of two wear mechanisms, abrasion and adhesion, was observed, resulting in a higher OCP and lower COF values.

## 4. Conclusions

In this work, the wear and friction properties of NiTi alloy in comparison with Ti-6Al-4V alloy processed by LENS, a laser beam directed energy deportation (LB-DED) method, were examined along with their corrosion behaviors. Within the limitations of this study and according to the obtained results, the following conclusions can be drawn:

- for the processing parameters used in this study, the LB-DED NiTi alloy exhibited better wear and corrosion performance as compared to the LB-DED Ti-6Al-4V alloy;
- a significant reduction in the wear volume loss of the LB-DED NiTi alloy was obtained as compared with the LB-DED Ti-6Al-4V alloy; however, the COF values in the case of the NiTi alloy processed by LENS were found to still be higher than the Ti-6Al-4V alloy;
- when no mechanical load is applied, the LB-DED Ti-6Al-4V alloy showed a slightly better corrosion behavior as compared to the LB-DED NiTi alloy;
- the drop observed on the OCP value at the beginning of the reciprocating sliding test (when the mechanical load was applied) was smaller for the LB-DED NiTi alloy as compared to the LB-DED Ti-6Al-4V alloy, which means that the NiTi alloy processed by LENS has a lower tendency to corrosion;
- both alloys have the repassivation ability after the sliding is ceased.

Our results showed that the additive manufacturing technology, such as LB-DED, can improve the wear and corrosion behavior of NiTi alloy. Even though the main drawback of NiTi alloy is the release of toxic ions of Ni when used in biomedical devices in vivo, the improvement in the wear and corrosion behavior of LB-DED NiTi alloy in the presence of PBS solution indicates its lesser susceptibility to the metallic ions release as compared to the commonly used Ti-6Al-4V alloy in biomedical applications.

## Figures and Tables

**Table 1:** Chemical composition of NiTi powder (provided by manufacturer).

Element	O	Al	C	Fe	H	N	Cr	Ni	Ti
Weight percent (%)	0.10	0.009	0.017	0.009	0.002	0.008	0.19	55.0	43.0

**Table 2:** Parameters used to produce NiTi and Ti-6Al-4V samples by LB-DED LENS process.

Material	Powder Feed Rate (g/s)	Traverse/Scan Speed (cm/s)	Laser Output Power (W)	Layer Thickness (mm)	Hatch Spacing (mm)
NiTi	0.06	8.47	280	0.02	0.02
Ti-6Al-4V	0.156	0.85, 1.27 or 1.69	350	0.02	0.02

## Data Availability

All data are available upon request. The data presented in this study are available on request from the corresponding author.
